# Supplementary material for: Joint Factor Performance Validity?—Network and Factor Structure of Performance Validity Measures in the Clinical Evaluation of Adult ADHD
Source: Behav Sci (Basel). 2025 Aug 15;15(8):1108. doi: 10.3390/bs15081108 (PMC12383783; doi:10.3390/bs15081108)
Supplement: Supplementary file 1 [file behavsci-15-01108-s001.zip › behavsci-3702391-supplementary.pdf]

Supplementary file

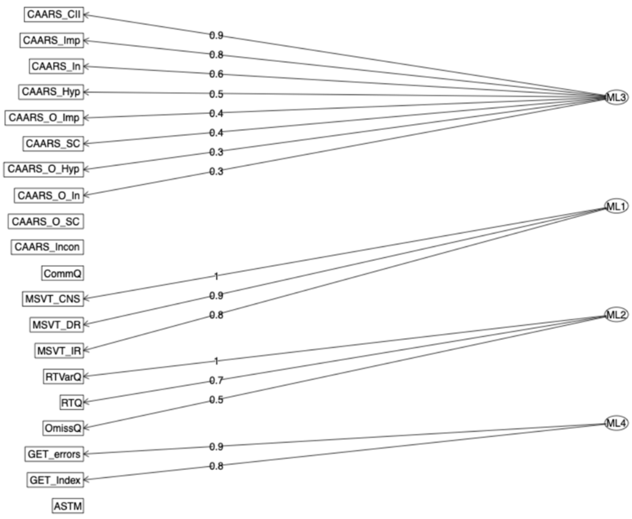

Figure S1. Exploratory Factor Analysis of PVT, CPT measures, ADHD symptoms (including SVTs) in the full sample (N=461)

Note. ASTM = Amsterdam Short Term Memory Test; MSVT_IR = Medical Symptom Validity Test (Immediate Recall); MSVT_DR = Medical Symptom Validity Test (Delayed Recognition); MSVT_CNS = Medical Symptom Validity Test (Consistency Scores); GET_Index = Groningen Effort Test (Index); GET_errors = Groningen Effort Test (Errors); RTQ= Reaction Time Q-score (Qb+); RTVarQ = Reaction Time Variation Q-score (Qb+); OmissQ= Omission Error Q-score (Qb+); CommQ= Comission Error Q-score (Qb+); RW_CAARS_Inattention = CAARS-L:S (Inattention); RW_CAARS_Hyperactivity = CAARS-L:S (Hyperactivity); RW_CAARS_Impulsivity = CAARS-L:S (Impulsivity); RW_CAARS_Self_Concept = CAARS-L:S (Self Concept); RW_CAARS_O_Inattention = CAARS-L:O (Inattention); RW_CAARS_O_Hyperactivity = CAARS-L:O (Hyperactivity); RW_CAARS_Impulsivity = CAARS-L:O (Impulsivity); RW_CAARS_O_Self_Concept = CAARS-L:O (Self Concept); CAARS_CII = CAARS-L:S (Infrequency Index); CAARS_Incon = CAARS-L:S (Inconsistency Index).

Factor loadings ≥ .30 indicate the significant relationships between the observed variables and their respective factors.

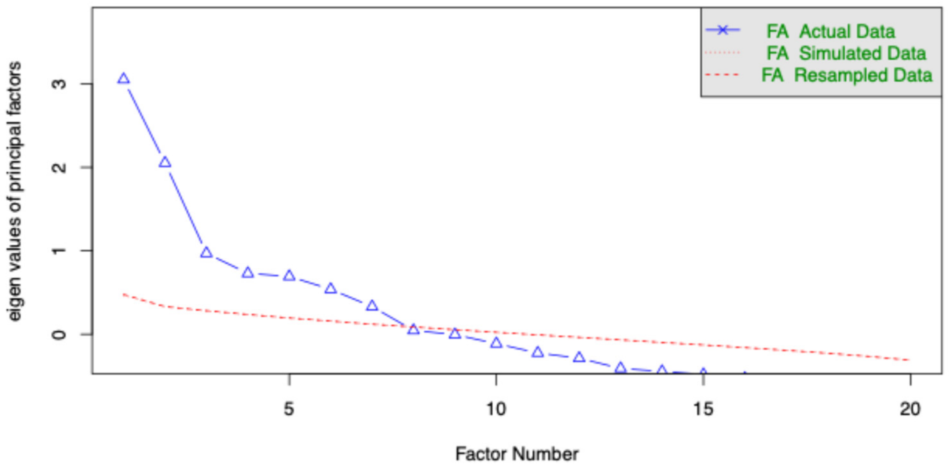

Figure S2. Scree plot following the exploratory factor analysis in the full sample (N=461)
